# Genetic diversity and recombination of bovine enterovirus strains in China

**DOI:** 10.1128/spectrum.02800-23

**Published:** 2024-02-05

**Authors:** Xiaoran Chang, Zhiyuan Zhang, Xuyuan Cui, Qun Zhang, Qian Lin, Junying Hu, Yidi Guo, Xinping Wang

**Affiliations:** 1 State Key Laboratory for Diagnosis and Treatment of Severe Zoonotic Infectious Diseases, Key Laboratory for Zoonosis Research of the Ministry of Education, Institute of Zoonosis, and College of Veterinary Medicine, Jilin University, Changchun, China; US Food and Drug Administration, USA

**Keywords:** bovine enterovirus, isolation, recombination, cattle, molecular analysis

## Abstract

Bovine enterovirus (BEV) consisting of enterovirus species E (EV-E) and F (EV-F) is the causative agent associated with respiratory and gastrointestinal diseases in cattle. Here, we reported the characterization, genetic diversity, and recombination of novel BEV strains isolated from the major cattle-raising regions in China during 2012–2018. Twenty-seven BEV strains were successfully isolated and characterized. Molecular characterization demonstrated that the majority of these novel BEV strains (24/27) were EV-E, while only few strains (3/27) were EV-F. Sequence analysis revealed the diversity of the circulating BEV strains such as species and subtypes where different species or subtype coinfections were detected in the same regions and even in the same cattle herds. For the EV-E, two novel subtypes, designated as EV-E6 and EV-E7, were revealed in addition to the currently reported EV-E1–EV-E5. Comparative genomic analysis revealed the intraspecies and interspecies genetic exchanges among BEV isolates. The representative strain HeN-B62 was probably from AN12 (EV-F7) and PS-87-Belfast (EV-F3) strains. The interspecies recombination between EV-E and EV-F was also discovered, where the EV-F7-AN12 might be from EV-E5 and EV-F1, and EV-E5-MexKSU/5 may be recombined from EV-F7 and EV-E1. The aforementioned results revealed the genetic diversity and recombination of novel BEV strains and unveiled the different BEV species or subtype infections in the same cattle herd, which will broaden the understanding of enterovirus genetic diversity, recombination, pathogenesis, and prevention of disease outbreaks.

**IMPORTANCE:**

Bovine enterovirus (BEV) infection is an emerging disease in China that is characterized by digestive, respiratory, and reproductive disorders. In this study, we first reported two novel EV-E subtypes detected in cattle herds in China, unveiled the coinfection of two enterovirus species (EV-E/EV-F) and different subtypes (EV-E2/EV-E7, EV-E1/EV-E7, and EV-E3/EV-E6) in the same cattle herds, and revealed the enterovirus genetic exchange in intraspecies and interspecies recombination. These results provide an important update of enterovirus prevalence and epidemiological aspects and contribute to a better understanding of enterovirus genetic diversity, evolution, and pathogenesis.

## INTRODUCTION

The *Enterovirus* genus of the family Picornaviridae is taxonomically grouped into 12 *Enterovirus* (A–L) and 3 *Rhinovirus* (A–C) species (1). These viruses share similar characteristics that are nonenveloped and icosahedral with the positive-stranded RNA genome (2, 3). They infect humans and animals and cause respiratory and gastrointestinal diseases (4–8). Bovine enterovirus (BEV) consists of two enterovirus species, the species E (EV-E) and F (EV-F) (7–9). EV-E and EV-F are separately divided into five subtypes (E1–E5) and seven subtypes (F1–F7) currently, respectively (3). The genome of enterovirus is approximately 7,100 to 7,450 nucleotides long, containing a single open-reading frame (ORF) that encodes a polyprotein. The polyprotein is later cleaved into three precursor proteins (P1, P2, and P3). The P1 protein is proteolytically processed into four structural viral proteins (VP1, VP2, VP3, and VP4), forming the viral capsid. The P2 precursor protein is cleaved to yield three nonstructural proteins (2A, 2B, and 2C). The P3 precursor protein is cleaved to generate four nonstructural proteins (3A, 3B, 3C, and 3D) (2, 3).

Recombination is a pervasive process generating diversity in most viruses, especially in *Enterovirus*. Recombination between serotypes for EVs was first observed in poliovirus in 1991 (10) and since then in a wide range of human enteroviruses. Among the members of *Enterovirus A*, the recombination occurred quite commonly in spite of lower recombination rates in EV-A71 in comparison with other subtypes in this species (11–13). Recombination rates in *Enterovirus B* are high, and a vast number of recombinant forms have been identified in this species (14–16). Recently, Luo et al. reported that BEV strains (GXNN2204 and GXGL2215) were, respectively, obtained from the genomic recombination of EV-E4 with EV-F3, and EV-E2 to EV-E4 (17). Recombination events in EVs are almost exclusively detected at the edges of the structural P1 region and within the nonstructural 5′UTR, P2, or P3 regions (14, 18). Although recombination has been shown to occur across the whole genome, not all recombinants may be viable. Thus, there would be selection of recombinants with breakpoints at particular locations (19). Since recombination is usually demonstrated by showing phylogeny inconsistencies (20, 21), the recombinant forms (RFs) can then be assigned based on clustering of the strains in the nonstructural region (16, 22), and breakpoints can be detected by performing bootscanning (21, 23). According to the criteria by the International Committee on Taxonomy of Viruses, members of species in the genus *Enterovirus* should have a high degree of amino acid identity (amino acid >70% in the polyprotein and amino acid >60% in P1) (1). In 1999, Oberste et al. found that the VP1 coding region carries major neutralization epitopes among the capsid proteins and is likely the optimal region for virus identification and molecular typing (24). Analysis of the VP1 sequences has been applied to genotype the most enteroviruses such as EV-A71 (11), CVA16 (25), CVA6 (26), and EV-E3 (27). Recently, Zell et al. suggested that enteroviruses were classified based on the amino acid sequence identities of the VP1 capsid protein, of which 70% to 85% were defined as heterologous types and >90% as homologous types (28).

In this study, we have isolated 27 strains of the BEV from cattle herds with diarrhea in various regions in China from 2012 to 2018, identified two novel EV-E subtypes in cattle herds in China, unveiled the coinfection of two enterovirus species and different subtypes in the same cattle herds, and revealed the enterovirus genetic exchange in intraspecies and interspecies recombination. These results provide an important update of the enterovirus prevalence and epidemiological aspects and contribute to a better understanding of enterovirus genetic diversity, evolution, and pathogenesis.

## RESULTS

### Isolation and characterization of the novel BEV strains

To isolate the potential BEV, the fecal samples collected from cattle herds with diarrhea in Jilin, Henan, and Ningxia provinces during 2012–2018 were processed. After infection, the Vero cells began to show cytopathic effects (CPEs) as early as 12 hours post-infection (hpi) and detached at 24–36 hpi compared to the uninfected cells (Fig. S1A and B). The isolates were further identified by RT-PCR with BEV-specific primers (5′UTR). After sequence analyses, the amplified fragments were turned out to contain the sequences of BEV. Information on these novel bovine virus strains is listed in Table 1. The TCID_50_ titers for the isolated BEV strains were determined to range from 10^2^/0.1 to 10^5.5^/0.1 mL (Fig. S1C).

**TABLE 1:** Molecular typing of enterovirus E and F isolates collected in China

Isolate	Location	EV type	Years	Isolation source	Host	Available sequence
HeN-A2	Henan	E	2018	Feces	Cattle	Complete genome
HeN-A3	Henan	E	2018	Feces	Cattle	Complete genome
HeN-B72	Henan	E	2018	Feces	Cattle	Complete genome
HeN-B78	Henan	E	2018	Feces	Cattle	Complete genome
HeN-B81	Henan	E	2018	Feces	Cattle	Complete genome
HeN-B83	Henan	E	2018	Feces	Cattle	Complete genome
HeN-B84	Henan	E	2018	Feces	Cattle	Complete genome
HeN-C4	Henan	E	2018	Feces	Cattle	Complete genome
JL-40	Jilin	E	2018	Feces	Cattle	Complete genome
JL-JY1	Jilin	E	2018	Feces	Cattle	Complete genome
JL-JY7	Jilin	E	2018	Feces	Cattle	Complete genome
JL-JY12	Jilin	E	2018	Feces	Cattle	Complete genome
JL-JY29	Jilin	E	2018	Feces	Cattle	Complete genome
JL-JY33	Jilin	E	2018	Feces	Cattle	Complete genome
JL-JY37	Jilin	E	2018	Feces	Cattle	Complete genome
JL-JY40	Jilin	E	2018	Serum	Cattle	Complete genome
JL-JY41	Jilin	E	2018	Feces	Cattle	Complete genome
JL-JY42	Jilin	E	2018	Feces	Cattle	Complete genome
NX-FY40	Ningxia	E	2018	Feces	Cow	Complete genome
NX-b-DR47	Ningxia	E	2018	Feces	Cattle	Complete genome
JL-HY61	Jilin	E	2013	Feces	Cattle	Complete genome
JL-HY68	Jilin	E	2012	Feces	Cattle	Complete genome
JL-DH13	Jilin	E	2012	Feces	Cattle	Complete genome
JL-GZL4	Jilin	E	2014	Feces	Cattle	Complete genome
HeN-A12	Henan	F	2018	Feces	Cattle	Complete genome
HeN-B62	Henan	F	2018	Feces	Cattle	Complete genome
HeN-YR91	Henan	F	2018	Feces	Cattle	Complete genome

### Decoding of the complete genome sequences for novel BEV strains

To decode the complete sequences for the novel BEV strains, a series of primers were designed individually for each strain and were used to amplify the genomic sequences (Table S1). After sequencing and joining the overlapped PCR-amplified fragments, the complete genomes for these viruses were obtained. Analyses showed that the lengths of complete genome sequences for these novel BEV strains varied from 7,433 to 7,470 nucleotides; the lengths of the 3′UTR were relatively diverse, ranging from 93 nucleotides to 136 nucleotides; and the lengths of the 5′UTR ranged from 810 nucleotides to 828 nucleotides with the relative conserved sequence. Additionally, the ORFs of these novel strains were highly conserved, ranging from 6,501 nucleotides to 6,531 nucleotides and encoding a polyprotein of 2,167–2,177 amino acids. Moreover, all strains shared a typical picornavirus genome organization. The newly identified viral strains and their GenBank accession numbers are listed in Table 2.

**TABLE 2:** The complete genome sequence of the newly identified virus strains

Strains	Complete genome (bp)	5'UTR (bp)	ORF (bp)	3'UTR (bp)	Accession no.
HeN-A2	7,459	810	6,531	118	MN598008
HeN-A3	7,453	814	6,525	114	MN598009
HeN-B72	7,457	815	6,525	117	MN598012
HeN-B78	7,453	812	6,525	116	MN598013
HeN-B81	7,457	815	6,525	117	MN598014
HeN-B83	7,451	812	6,525	114	MN598015
HeN-B84	7,469	819	6,525	125	MN598016
HeN-C4	7,469	821	6,525	123	MN598017
JL-40	7,434	816	6,525	93	MN598019
JL-JY1	7,453	818	6,525	110	MN598024
JL-JY7	7,455	816	6,525	114	MN598025
JL-JY12	7,444	814	6,525	102	MN598026
JL-JY29	7,450	815	6,525	107	MN598027
JL-JY33	7,454	813	6,525	116	MN598028
JL-JY37	7,465	814	6,525	126	MN598029
JL-JY40	7,457	814	6,525	118	MN598030
JL-JY41	7,461	814	6,528	119	MN598031
JL-JY42	7,467	815	6,525	127	MN598032
NX-FY40	7,470	817	6,531	122	MN598039
NX-b-DR47	7,449	810	6,525	114	MN598037
JL-HY61	7,462	816	6,531	115	MN598022
JL-HY68	7,463	828	6,531	104	MN598023
JL-DH13	7,451	817	6,531	103	MN598020
JL-GZL4	7,447	815	6,528	104	MN598021
HeN-A12	7,433	822	6,504	107	MN598010
HeN-B62	7,458	821	6,501	136	MN598011
HeN-YR91	7,460	825	6,504	131	MN598018

### The new BEV strains shared high sequence identity with EV-E and EV-F reference strains

To determine the sequence identity of these novel BEV strains with the reference strains, a comprehensive analysis of the nucleotide and amino acid sequences of VP1, P1, and 2C + 3 CD was performed. As given in Table 3, five strains, namely, HeN-A2, NX-FY40, JL-HY61, JL-HY68, and JL-DH13, shared the nucleotide sequence identity of 80.3%–89.1% and amino acid sequence identity of 96.5%–97.5% with the VP1 of EV-E3 reference strain HY12 (KF748290), indicating that these five isolated strains are evolutionarily closer to EV-E3 strains. In Table 3, JL-JY12, JL-JY29, JL-JY41, and JL-GZL4 strains shared a relatively high nucleotide and amino acid sequence identity with the VP1 of the EV-E1 reference strain VG-5–27 (NC_001859), respectively, varying from 79.8% to 80.9% and 94.3% to 96.1%, indicating that these four isolated strains belong to the EV-E1 strains. HeN-B78, HeN-B84, and HeN-C4 strains from Henan province shared a relatively high nucleotide identity of 73.6% to 75.6% and amino acid sequence identity of 91.4% to 94.3% with the VP1 of EV-E2 reference strain PS 42 (DQ092792), suggesting they are close to EV-E2 strains. The strain HeN-B62 shared the highest nucleotide and amino acid sequence identity with VP1 of EV-F3 strain PS-87-Belfast (DQ092794), 76.9% and 92.7%, respectively. HeN-A12 and HeN-YR91 strains shared a relatively high nucleotide and amino acid sequence identity with VP1 of the EV-F1 strain BEV-261 (DQ092770), varying from 75.5% to 81.3% and 89.8% to 97.5%, respectively, indicating that they were evolutionarily closer to EV-F1 strains. Interestingly, twelve strains, namely, JL-40, JL-JY1, JL-JY7, HeN-B72, HeN-B81, HeN-B83, JL-JY33, JL-JY37, JL-JY40, JL-JY42, HeN-A3, and NX-b-DR47, shared low nucleotide and amino acid identity with all of the EV-E strains, with the highest only 88.6% amino acid identity with EV-E2 strain PS 42 (DQ092792), indicating that those strains likely belong to a new subtype (Table S3). Taken together, the aforementioned results indicate these novel BEV strains are either close to EV-E or EV-F based on their sequence identities.

**TABLE 3:** Percent identity of nucleotide and amino acid sequences between isolated strains and enterovirus reference strains

Strains	Reference enterovirus strains	Identity (%)						Subgenotype
		VP1		P1		2C + 3 CD		
		Nt	Aa	Nt	Aa	Nt	Aa	
HeN-A2	HY12 (KF748290)	80.3	96.5	81.1	96.2	82.5	96.8	EV-E3
NX-FY40	HY12 (KF748290)	82.6	97.5	82.8	97.4	83.3	97	EV-E3
JL-HY61	HY12 (KF748290)	89.1	97.5	88.5	98.1	84.5	96.8	EV-E3
JL-HY68	HY12 (KF748290)	88.8	97.5	88.7	97.9	84.3	96.7	EV-E3
JL-DH13	HY12 (KF748290)	88.5	97.5	88.5	98.1	84.4	96.9	EV-E3
JL-JY12	VG-5–27 (NC_001859)	80.9	96.1	80.4	97	79.4	95.6	EV-E1
JL-JY29	VG-5–27 (NC_001859)	79.8	94.3	79.1	96.4	79.6	95.7	EV-E1
JL-JY41	VG-5–27 (NC_001859)	80	95.4	79.3	96.5	79.2	96.1	EV-E1
JL-GZL4	VG-5–27 (NC_001859)	80.7	95	79.8	96.7	81	97.6	EV-E1
HeN-B78	PS 42 (DQ092792)	73.9	91.8	76.3	93.9	80.4	96.3	EV-E2
HeN-B84	PS 42 (DQ092792)	73.6	91.4	76.4	94.2	79.2	95.1	EV-E2
HeN-C4	PS 42 (DQ092792)	75.6	94.3	76.9	95.8	81.1	96.9	EV-E2
JL-40	PS 42 (DQ092792)	73.3	87.9	76.2	91.9	78.9	95.1	EV-E7
JL-JY1	PS 42 (DQ092792)	73.3	87.9	76.4	92.1	78.7	95.1	EV-E7
JL-JY7	PS 42 (DQ092792)	73.3	87.9	76.3	91.7	78.9	95.1	EV-E7
HeN-B72	PS 42 (DQ092792)	73.1	88.6	75.7	91.8	80.2	96.5	EV-E7
HeN-B81	PS 42 (DQ092792)	72.9	87.1	75.5	91.4	79.5	95.1	EV-E7
HeN-B83	PS 42 (DQ092792)	73.1	87.5	75.6	91.4	80.4	95.6	EV-E7
JL-JY33	PS 42 (DQ092792)	73.3	88.2	75.7	92.3	78.8	95.7	EV-E7
JL-JY37	PS 42 (DQ092792)	73.1	88.2	75.4	92.1	78.7	95.7	EV-E7
JL-JY40	PS 42 (DQ092792)	73.1	88.2	75.4	2.1	78.6	95.3	EV-E7
JL-JY42	PS 42 (DQ092792)	73	87.9	75.6	92	78.8	95.7	EV-E7
HeN-A3	PS 42 (DQ092792)	72.9	85	75.6	90.3	78.1	92.8	EV-E6
NX-b-DR47	PS 42 (DQ092792)	72.1	85.4	75.1	90.3	77.7	92.3	EV-E6
HeN-B62	PS-87-Belfast (DQ092794)	76.9	92.7	76.9	93.8	82	97.7	EV-F3
HeN-A12	BEV-261 (DQ092770)	81.3	97.5	81	97.5	83	97.8	EV-F1
HeN-YR91	BEV-261 (DQ092770)	75.5	89.8	75.5	90.5	80.9	95.1	EV-F1

### Phylogenetic analyses clustered the novel BEV strains to EV-E and EV-F species

**Fig 1:**
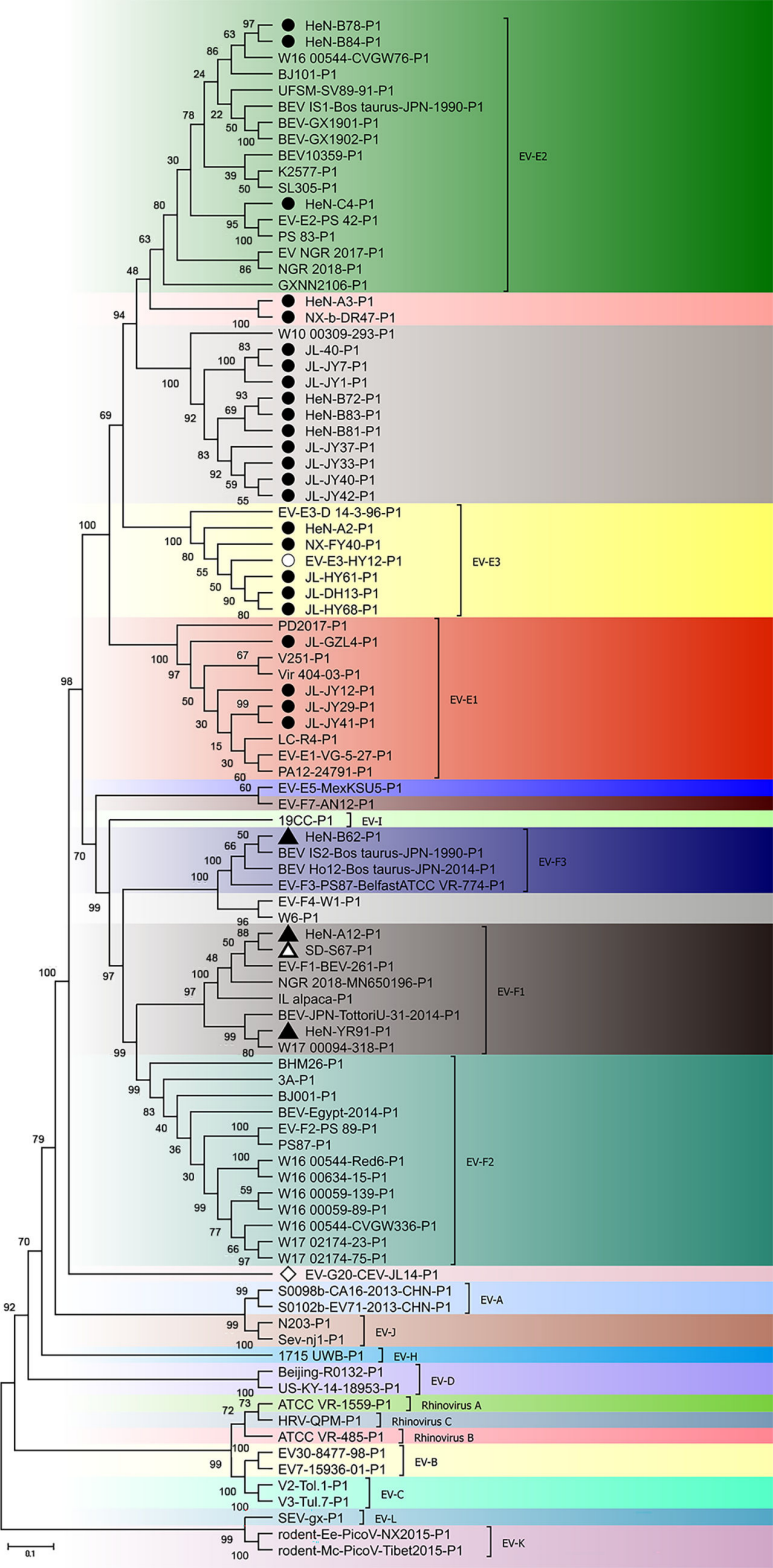
Phylogenetic analyses of P1 on the novel bovine enterovirus strains. The reference sequences include the representative sequences of all known EV-E and EV-F types and the representative sequences of all 12 enterovirus species and 3 rhinovirus species in the *Enterovirus* genus were used as outgroup sequences. The amino acid sequences of P1 were used to construct the phylogenetic tree using the maximum likelihood method with 1,000 bootstrap replications. Bootstrap values of >50 are shown at the nodes. The scale bar represents 10% nucleotide sequence divergence for maximum likelihood methods. The analysis models for ML methods for the P1 amino acid are LG + G + I. Viruses are marked with symbols as follows: ● refers to the EV-E strains obtained in this study; ▲ refers to the EV-F strains obtained in this study; ◇ stands for the CEV-JL14 strain; ○ refers to the HY12 EV-E strain isolated from cattle; △ stands for the SD-S67 strain (an EV-F) isolated from goats.

**Fig 2:**
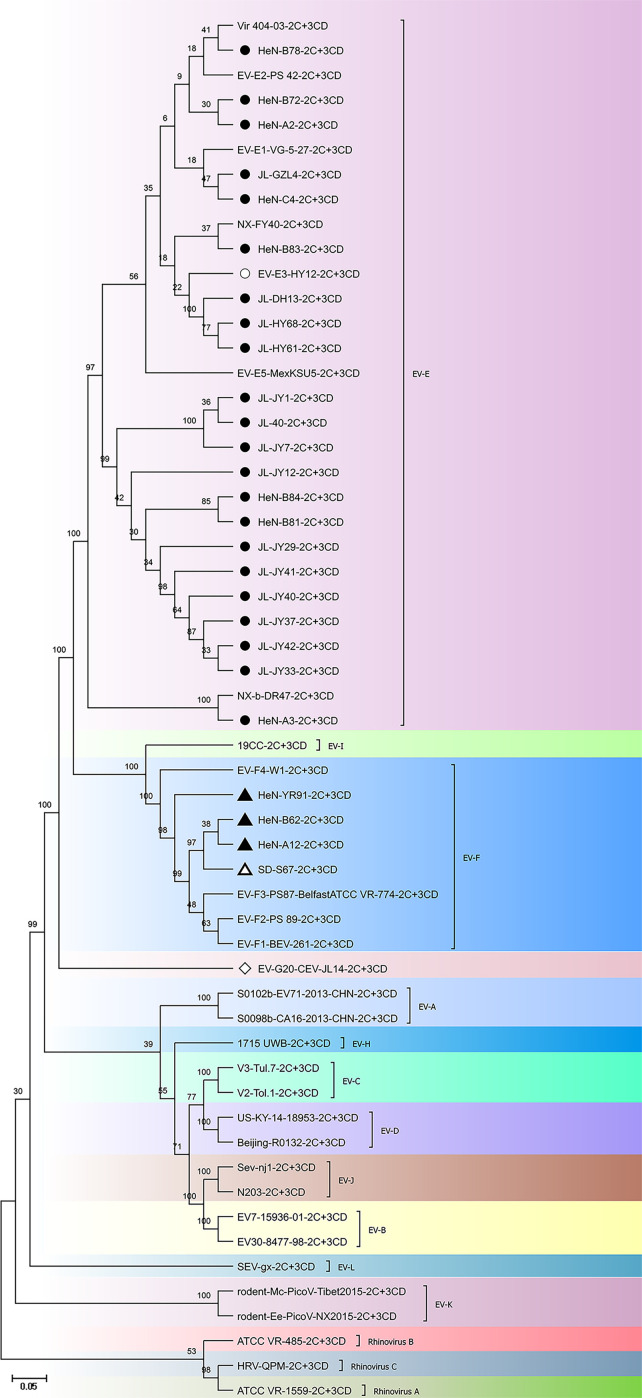
Phylogenetic analyses of 2C + 3 CD on novel bovine enterovirus strains. The reference sequences include the representative sequences of all known EV-E and EV-F types and the representative sequences of all 12 enterovirus species and 3 rhinovirus species in the *Enterovirus* genus were used as outgroup sequences. The amino acid sequences of 2C + 3 CD were used to construct the phylogenetic tree using the maximum likelihood method with 1000 bootstrap replications. Bootstrap values of >50 are shown at the nodes. The scale bar represents 5% nucleotide sequence divergence for maximum likelihood methods. The analysis models for ML methods for the 2C + 3 CD amino acid is LG + G + I. Viruses are marked with symbols as follows: ● refers to the EV-E strains obtained in this study; ▲ refers to the EV-F strains obtained in this study; ◇ stands for the CEV-JL14 strain; ○ refers to the HY12 EV-E strain isolated from cattle; △ stands for the SD-S67 strain (an EV-F) isolated from goats.

To further confirm the phylogenetic status of these novel bovine enterovirus strains, phylogenetic analysis was performed based on the amino acid sequences of P1 and 2C + 3 CD using the maximum likelihood method. The representative sequences of all known types of EV-E and EV-F, and the representative sequences of all 12 enterovirus species and 3 rhinovirus within the *Enterovirus* genus were used as outgroup sequences. The HY12 virus was used as a reference strain of EV-E3 for 2C + 3 CD phylogenetic tree analyses since it is the only EV-E3 virus strain with 2C + 3 CD sequences available. As shown in Fig. 1 and 2, 24 isolated strains were clustered in the same clade as EV-E, and 3 isolated strains, namely, HeN-A12, HeN-B62, and HeN-YR91, belong to the branch of EV-F strains. These results were congruent with the species and subtypes determined based on sequence identity, revealing the inconsistency of phylogenetic clustering between those of P1 and 2C + 3 CD.

### Two novel EV-E subtypes revealed for bovine enterovirus

**Fig 3:**
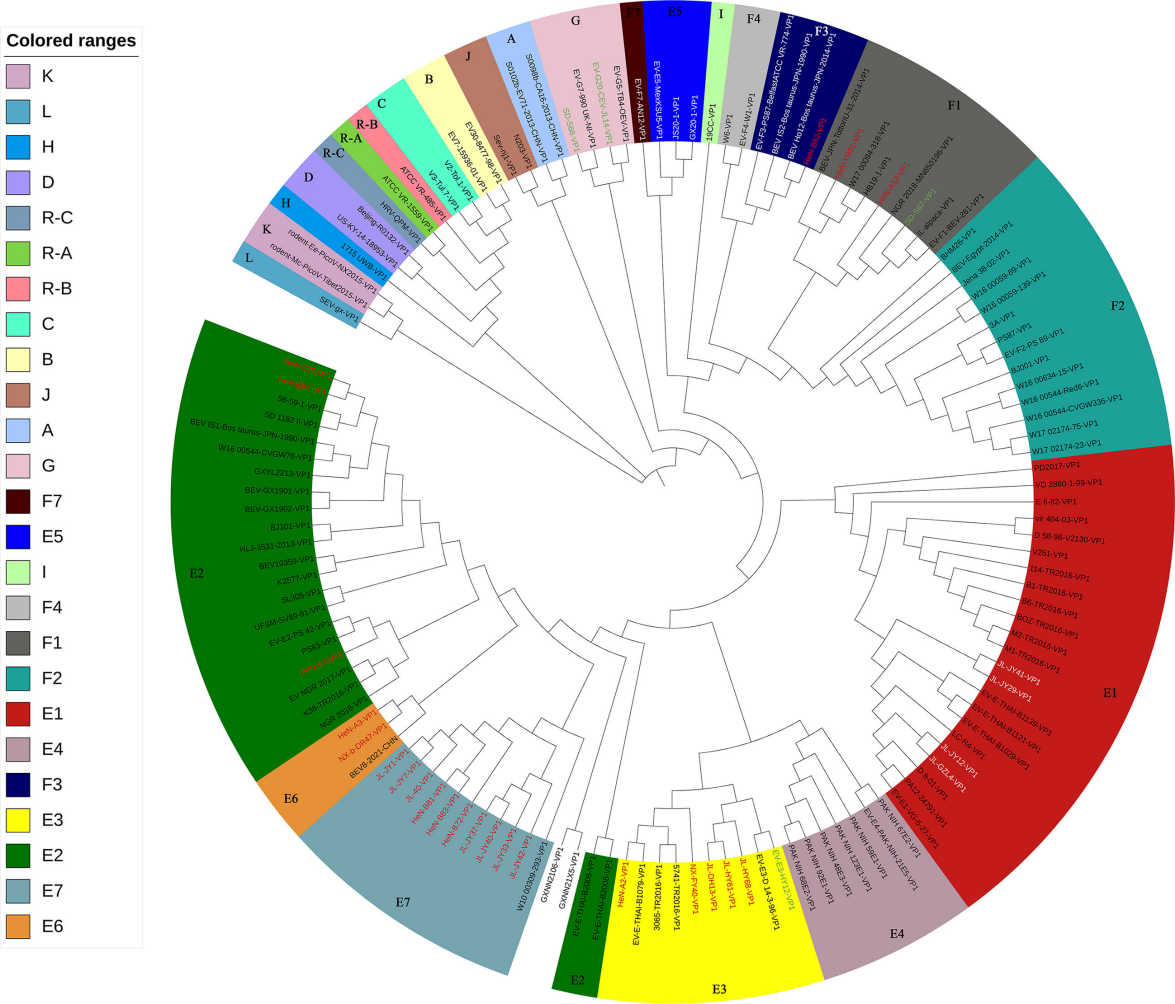
Phylogenetic analyses for BEV subtyping. Phylogenetic analysis was performed to subtype the BEV strain based on the VP1 amino acid sequences. The reference sequences including the representative sequences of all known EV-E and EV-F types and the representative sequences of all 12 enterovirus species and 3 rhinovirus species within the *Enterovirus* genus were used as outgroup sequences. A phylogenetic tree was generated using the maximum likelihood method with 1000 bootstrap replications. The scale bar represents 10% nucleotide sequence divergence for maximum likelihood methods. The analysis model for ML methods for the VP1 amino acid is LG + G. Viruses are marked with symbols as follows: red characters refer to the EV-E and EV-F strains obtained in this study, and white characters in the EV-E1 subtype refer to the EV-E strains obtained in this study; the green character stands for the strains isolated in the laboratory. The subtypes of the strains are shown in the figure. R-A stands for rhinovirus A, R-B stands for rhinovirus B, R-C stands for rhinovirus C.

To determine the subtypes for the novel BEV strains, phylogenetic analyses were further performed for these EV-E and EV-F strains based on the sequence identity of VP1. Twenty-four novel EV-E strains were clustered separately into five subtypes. The strains JL-JY12, JL-JY29, JL-JY41, and JL-GZL4 were clustered to the same subtype as VG-5–27 (EV-E1), sharing 94.3%–96.1% (Table 3) VP1 amino acid sequence identity. The strains HeN-B78, HeN-B84, and HeN-C4 were clustered to the same subtype with PS 42, an EV-E2 since they shared the VP1 amino acid sequence identity of 91.4%–94.3% (Table 3). The strains HeN-A2, NX-FY40, JL-HY61, JL-HY68, and JL-DH13 were clustered to the same type as HY12 (EV-E3), where they shared 96.5%–97.5% VP1 amino acid sequence identity (Table 3). In addition to the aforementioned three subtypes (EV-E1–EV-E3), two novel subtypes excluding EV-E4 and EV-E5 were identified (Fig. 3). The first new subtype contained the strains HeN-A3 and NX-b-DR47. They had the VP1 amino acid sequence identity of 97.5% of each other, with VP1 amino acid sequence identity of 50.7%–85.4% from all other EV-E reference strains (Table S3). These results demonstrated that HeN-A3 and NX-b-DR47 belong to a new subtype, designated as EV-E6. The second novel subtype consisted of JL-40, JL-JY1, JL-JY7, HeN-B72, HeN-B81, HeN-B83, JL-JY33, JL-JY37, JL-JY40, and JL-JY42 strains, with the identity of 96.1%–100% in the VP1 amino acid sequence among them (Table S3). These strains presented 50.4%–88.6% amino acid identity on the VP1 with other EV-E reference strains (Table S3), indicating they are also a new subtype, designated as EV-E7.

Similarly, analysis on the subtypes for EV-F showed that HeN-A12 and HeN-YR91 strains were clustered to the same subtype as BEV-261 (EV-F1), with 89.8%–97.5% amino acid sequence identity of VP1 (Table 3). The strain HeN-B62 was clustered to the subtype as PS-87-Belfast (EV-F3), with 92.7% amino acid sequence identity (Table 3).

### Different subtypes and species of enterovirus detected in the same cattle herd

Further analysis on the enterovirus species showed that the HeN-B78, HeN-B84, and HeN-B62 strains from the same cattle herd were clustered to different enterovirus species, where HeN-B78 and HeN-B84 strains were grouped to EV-E and HeN-B62 clustered to EV-F. These results clearly demonstrated the mixed infections of EV-E and EV-F simultaneously in the same cattle herd. Similarly, HeN-A12 and HeN-A3 strains from another cattle herd also belonged to different species, where HeN-A3 was grouped to EV-E and HeN-A12 was grouped to EV-F. These results demonstrated the unusual coinfection of EV-E and EV-F in the cattle herd.

It is interesting to note that different BEV subtype infections were also revealed in the same cattle herds. As shown in Fig. 1 and 3, JL-JY12, JL-JY29, and JL-JY7; HeN-A2 and HeN-A3; HeN-B78, and HeN-B84 and HeN-B81 from the same herds were clustered to different subtypes. These results demonstrated the complexity of BEV infection.

### Recombination detected in the novel isolated bovine enterovirus strains

**Fig 4:**
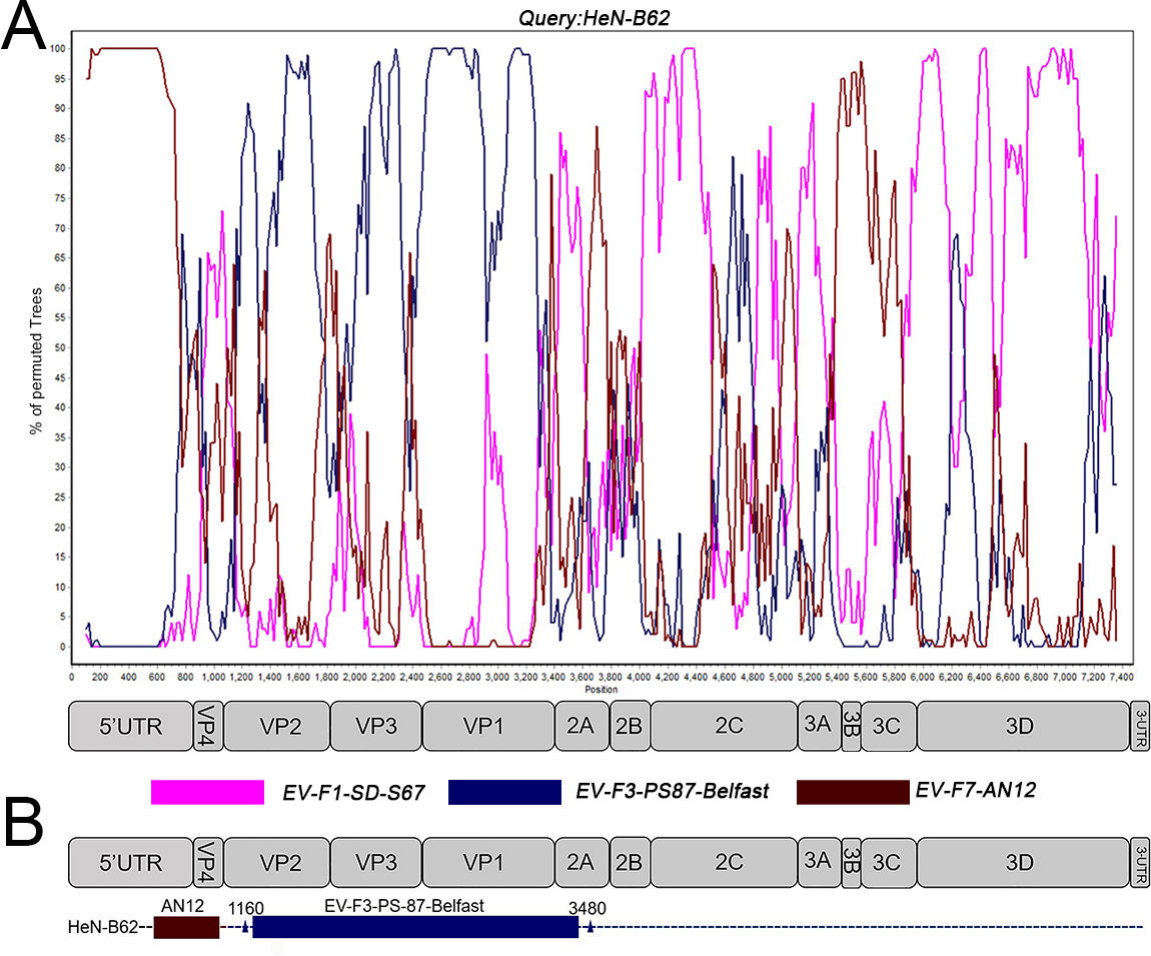
Recombination analyses of the HeN-B62 strain with EV-F types. The genome sequence of HeN-B62 (**A**) was used as query sequences in the bootscan analysis. The structural map of the bovine enterovirus genome is displayed below each panel. The possible crossover breakpoints were marked with a rectangular box with the enterovirus subtypes and represented by corresponding colors (**B**). Each point plotted is the percentage identity within a sliding window 200 nt wide centered on the position plotted, with a step size of 20 nt between points.

To characterize the genomic features and explore the origin of the isolated strains, recombination analyses were performed using SimPlot software and the RDP4 package with multiple algorithms. The *P* values by the individual tools implemented in the RDP are shown in Table S4. The complete genome sequences of 24 isolated EV-E and 3 EV-F strains were selected as the query sequences for similarity and bootscan analysis with EV-E and EV-F reference sequences, respectively. As shown in Fig. 4A and B, the HeN-B62 strain resembled the EV-F7-AN12 strain in the sequence before the VP2 junction (nearby 1,160 bp), while it was more similar to the EV-F3-PS-87-Belfast strain than other strains for the fragment region (1,160–3,480 bp). Additionally, HeN-B62 contained an unidentified sequence that was apparently not related to the PS-87-Belfast strain in the fragment region (3,480–7,400 bp). Therefore, two recombination events possibly occurred in the HeN-B62 strain: one occurred between EV-F7-AN12 and EV-F3-PS-87-Belfast strains, with the breakpoint located in the VP2 gene, and the other occurred between EV-F3-PS-87-Belfast and unidentified sequences, with one breakpoint located in the 2A gene (Fig. 4B). The HeN-A12 (EV-F1) strain showed the highest degree of similarity to the EV-F1-SD-S67 strain in the P1 region. However, it contained an unidentified sequence that was apparently not related to the SD-S67 strain in the P2 and P3 regions (Fig. S2A). No apparent recombination was found in the HeN-YR91 strain with EV-F (data not shown). The HeN-A2 strain showed the highest degree of similarity to HY12 (EV-E3) in the P1 region. However, it contained an unidentified sequence that was apparently not related to the HY12 strain in the P2 and P3 regions (Fig. S2B). No apparent recombination events were found in other isolated EV-E strains (data not shown).

### Interspecies and intraspecies recombination revealed on bovine enterovirus strains

**Fig 5:**
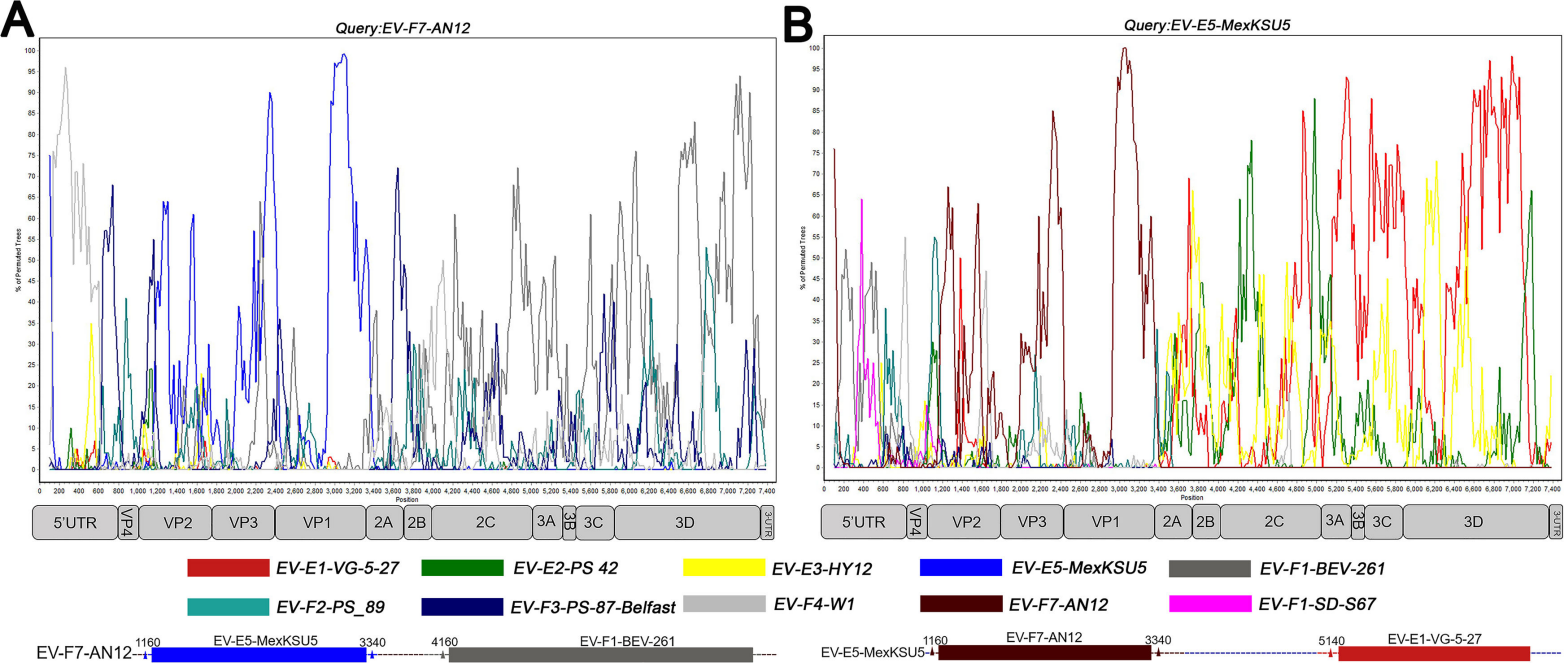
Recombination analyses between EV-E and EV-F types. The genome sequences of EV-F7-AN12 (**A**) and EV-E5-MexKSU/5 (**B**) were used as query sequences in the bootscan analysis. The structural map of the bovine enterovirus genome was displayed below each panel. The possible crossover breakpoints were marked with a rectangular box with the enterovirus subtypes and represented by corresponding colors. Each point plotted is the percentage identity within a sliding window 200 nt wide centered on the position plotted, with a step size of 20 nt between points.

To characterize the genomic features of the predominant EV-E and EV-F strains, the complete genome sequences of EV-E1-EV-E3, and EV-E5; EV-F1-EV-F4, and EV-F7 standard strains; and VG-5–27, PS 42, HY12, MexKSU/5, BEV-261, PS-89, PS-87-Belfast, W1, and AN12 were selected as the query sequences for similarity and bootscan analysis (Fig. 5). As shown in Fig. 5A, the interspecies recombination between EV-E and EV-F was analyzed. Bootscan analyses indicated that EV-F7-AN12 might be a recombinant virus between EV-E5-MexKSU/5 and EV-F1-BEV-261 strains, with one breakpoint located at the VP1/2A junction (3,340 bp). EV-F7-AN12 shared the highest similarity to the EV-E5-MexKSU/5 strain in the sequence region (1,160–3,340 bp), while it had the highest similarity to the EV-F1-BEV-261 strain in the sequence after 4,160 bp. As observed in Fig. 5B, the possible recombination events occurred with at least three recombinant breakpoints on EV-E5-MexKSU/5, corresponding to the VP4/VP2 junction, VP1/2A junction, and 3A/3B junction. The EV-E5-MexKSU/5 strain contained alternative regions between the VP1/2A and 3A/3B junctions that were closely related to EV-E3-HY12 and EV-E2-PS 42 strains. EV-E5-MexKSU/5 shared the highest similarity to the EV-F7-AN12 strain within the sequence region (1,160–3,340 bp), while it had the highest similarity to the EV-E1-VG-5–27 strain in the sequence after 5,140 bp. These results demonstrated the interspecies recombination among bovine enteroviruses.

The intraspecies recombination among different EV-F subtypes was also analyzed. For the EV-F1-BEV-261 strain (Fig. S3A), it had the similar sequence as that of the EV-E5-MexKSU/5 strain before the 5′UTR junction (nearby 700 bp), while it was closer to EV-F2-PS_89 in the fragment region (700–4,100 bp). Additionally, EV-F1-BEV-261 shared the highest similarity to the EV-F7-AN12 strain in the sequence after 4,100 bp. For the EV-F2-PS_89 strain (Fig. S3B), the fragment (1,160–3,920 bp) was more similar to that of EV-F1-BEV-261, and the subsequent sequence (3,920–7,400 bp) shared the highest similarity to the EV-F3-PS-87-Belfast strain. As for EV-F3-PS-87-Belfast (Fig. S3C), the fragment (1,140–3,940 bp) was more similar to that of EV-F4-W1, and the subsequent sequence (3,940–7,400 bp) shared the highest similarity to EV-F2-PS_89 (Fig. S3C). The fragment (1,160–3,840 bp) of EV-F4-W1 was more similar to that of EV-F3-PS-87-Belfast (Fig. S3D).

## DISCUSSION

In this study, we have isolated 27 novel bovine enteroviruses from the major cattle-raising regions during 2012–2018 in China, explored the genetic diversity and recombination of the novel BEV strains, unveiled two new subtypes of EV-E designated as EV-E6 and EV-E7, and revealed the different BEV species or subtype infection on the same cattle herds.

BEV is the causative agent associated with an emerging disease in China, which leads to an important economic loss to the cattle industry. Based on virus genetic variability and molecular difference, BEV is divided into two species (genotype or serotype), EV-E and EV-F, where EV-E and EV-F are further divided into five subtypes (subgenotype) and seven subtypes, respectively (3). Although EV-E and EV-F infections were reported in China in the early 2010s (9, 27), they remain largely unknown on many aspects such as the molecular difference, the virus genetic diversity and origin, the geographical distribution, and the transmission pattern. To unveil the underlying aspects of BEV infections, we performed BEV isolation using the representative specimens detected as BEV-positive by ELISA methods (29). Molecular characterization demonstrated that 27 BEV strains were successfully obtained. Sequencing and analyses of these BEV strains revealed that the majority of them are EV-E, suggesting that the predominant BEV infections are caused by EV-E. Interestingly, we found that different subtype strains within EV-E or EV-F were isolated from the same cattle herds; even EV-E (HeN-B78 and HeN-B84) and EV-F (HeN-B62) were both revealed in the same cattle herds. These results clearly demonstrated the coinfection of enterovirus species or subtypes. Since mutation and recombination extensively occurred among different BEV genotypes, the emergence and importation of new genotypes or subtypes of BEV should be continuously monitored. The findings that the same subtypes were revealed in different regions or provinces indicate that BEV may be transmitted between different regions by live cattle distribution. More importantly, we found that some of these newly identified strains are likely the recombinants resulting from either enterovirus intraspecies or interspecies recombination, which enriches the understanding of underlying enterovirus pathogenicity and evolution.

The amino acid sequence identity of VP1 is currently considered the standard for enterovirus classification (30). Recently, according to the suggestion by Zell et al., enteroviruses were classified based on the amino acid sequence identities of the VP1 capsid protein, of which 70% to 85% were defined as heterologous types and >90% as homologous types (28). Based on the criteria for enterovirus demarcation and our analyses on the available EV-E sequences, we proposed that the EV-E strains should be divided into seven subtypes. In addition to the five current reported subtypes EV-E1–EV-E5, obviously two novel subtypes for EV-E strains were discovered. We designated them as EV-E6 and EV-E7. This finding will broaden our current knowledge on enterovirus classification.

Genetic recombination plays important roles in the genetic variation and evolution of viruses. Although EV-A71 was mostly selected for recombination analysis in previous studies on *Enterovirus*, there were few studies on recombination analysis of bovine enterovirus (31–33). In the present study, the complete genome sequences of the novel BEV strains were used as the query sequences. Comprehensive recombination analyses revealed two recombination events that occurred possibly in the HeN-B62 strain. One recombination event occurred between EV-F7-AN12 and EV-F3-PS-87-Belfast strains, with the breakpoint located in the VP2 gene, and the other occurred between EV-F3-PS-87-Belfast and unidentified sequences, with the breakpoint located in the 2A gene, suggesting an intraspecies recombination. More importantly, interspecies recombination between EV-E and EV-F was found, where the EV-F7-AN12 strain is likely a recombinant from EV-E5 and EV-F1, and EV-E5-MexKSU/5 may be a recombinant from EV-F7 and EV-E1. It was also found that there was intraspecific recombination between the EV-F strains. We found that recombination breakpoints in the *Enterovirus* genome were frequently located in the nonstructural regions 5′UTR, P2, and P3 but rarely in the structural region P1, which is consistent with previous reports (34). The discovery that the nonstructural regions P2 and P3 act as the hot spots for recombination in BEV may be due to the higher homology of P2 and P3 regions than the structural region shared by BEV strains. Moreover, the inconsistent topology of phylogenetic trees suggested that it was not sufficient to genotype the recombinant viruses by VP1 alone.

The EV-F strains were first isolated in Northeast China in 2011. Since the discovery of the EV-E strain in 2012 in China, many BEV infections were reported. Our analyses on BEV strains demonstrated the EV-E strains as the predominant genotype in China. Since mutation and recombination extensively occurred among different BEV genotypes, the emergence and importation of new genotypes or subtypes of BEV should be continuously monitored (9, 27, 30).

## MATERIALS AND METHODS

### Sample collection and detection

A total of 2,721 fecal swabs collected from Jilin, Ningxia, and Henan were processed as previously described (27). All the samples were shipped on ice and then stored at −80°C.

### Cell culture and virus isolation

Vero cells were cultured in Dulbecco’s modified Eagle medium (DMEM) (Invitrogen, Carlsbad, CA, USA) supplemented with 5% fetal bovine serum (HyClone, Beijing, China), 2 µg/mL gentamycin, and 2 mM L-glutamine (Invitrogen). Virus isolation was performed as previously described (27). Briefly, the fecal swabs were diluted at 1:10 (W/V) in a 10 mM phosphate-buffered saline (PBS) (pH 7.4) and clarified by low-speed centrifugation (3,000 × *g* for 10 min). The supernatants were filtered through 0.45-µm pore membrane filters (JIN TENG, Tianjin, China). Virus isolation was performed on these specimens using Vero cells. The CPE was captured using a Canon digital camera (Canon, Tokyo, Japan). The noninfected cells were used as negative controls.

### TCID_50_ titration

TCID_50_ titration of the isolated strains was performed using 96-well plates following the procedure previously described (27). Briefly, the viruses were diluted at 10 × serial dilutions and used to infect the cells for each dilution. The cytopathic effects on the Vero cells were observed and counted at 48 hpi. TCID_50_ was calculated following a standard procedure based on the Reed–Muench method (35).

### RNA extraction and PCR

Viral RNA was extracted from the sample using the TRIzol reagent (Invitrogen) as previously described (27). cDNA was synthesized by reverse transcription using the Bio RT-cDNA kit (Invitrogen) following the manufacturer’s instructions. The primers for amplification of the complete genome sequence were designed based on the variation of each strain (Table S1). cDNA ends were determined using the FirstChoice RLM-RACE kit (Thermos Fisher Scientific, Carlsbad, CA). PCR products were subjected to electrophoresis using 1.0% agarose gel and purified for DNA sequencing (Sangon Biotechnology, Shanghai, China). The resulting sequences were analyzed using the Basic Local Alignment Search Tool (BLAST). The complete genome sequences were assembled using Lasergene software (version 7.0, DNASTAR, Madison, WI, USA).

### Data collection and nucleotide sequence data sets

Genome sequences for enteroviruses from species E and F were retrieved from the GenBank database (to March 2023) (Table S2). Wrongly annotated sequences and coding sequences (CDSs) containing internal termination codons were discarded. A sequence alignment including the complete genomes of enterovirus strains was generated with Clustal W implemented in BioEdit, version 7.2.3, software (http://www.mbio.ncsu.edu/bioedit/bioedit.html) and split into three partitions corresponding to the VP1, P1, and 2C + 3 CD genomic regions.

### Maximum likelihood phylogenetic analysis and recombination analysis

Maximum likelihood trees were generated with MEGA-7 software (36) using the best evolutionary model and 1,000 bootstrap replicates for the estimation of node consistency (37). The merged sequence file (2C + 3 CD) was first aligned by MEGA-7 with the Clustal W method to generate nucleotide alignment. Phylogenetic trees were visualized by using the Interactive Tree of Life version 6 (https://itol.embl.de/).

Recombination analyses were performed using a recombination detection software package (RDP 4). A total of seven methods implemented in RDP4 were applied: RDP, Bootscan, GENECONV, 3Seq, Chimaera, MaxChi, and SiScan. Recombination detected by at least three of the seven methods with a *P* value cutoff 0.05 was considered true recombination. The similarity plot and bootscan analysis were performed by SimPlot 3.5.1 (JHK University, Baltimore, MD, USA). The pairwise similarity between all sequences in multiple sequence alignment was calculated with a 200-nt window moved along the sequence in 20-nt steps.

## Data Availability

All nucleotide sequence data are available in GenBank under accession numbers MN598008-MN598032, MN598037, and MN598039.

## SUPPLEMENTAL MATERIAL

Fig. S1 spectrum.02800-23-s0001.tif Cytopathic effects (CPE) and growth characteristics analysis in Vero cells.

Fig. S2 spectrum.02800-23-s0002.tif Recombination analyses of HeN-A12 and HeN-A2 strains with EV-E and EV-F types.

Fig. S3 spectrum.02800-23-s0003.tif Recombination analyses between EV-F subtypes.

Legends spectrum.02800-23-s0004.docx Supplemental figure legends.

Table S1 spectrum.02800-23-s0005.docx Primer sequences to amplify full-length sequences.

Table S2 spectrum.02800-23-s0006.docx Reference strains for enterovirus.

Table S3 spectrum.02800-23-s0007.xlsx Percent identities of amino acid sequence in VP1 of bovine enterovirus strains compared with reference strains of EV-E and EV-F.

Table S4 spectrum.02800-23-s0008.docx Algorithms of the RDP4 package used to predict the recombination event.

ASM does not own the copyrights to Supplemental Material that may be linked to, or accessed through, an article. The authors have granted ASM a non-exclusive, world-wide license to publish the Supplemental Material files. Please contact the corresponding author directly for reuse.
