# Supplementary figures and images for: Genetic diversity and recombination of bovine enterovirus strains in China
Source: Microbiol Spectr. 2024 Feb 5;12(3):e02800-23. doi: 10.1128/spectrum.02800-23 (PMC10913430; doi:10.1128/spectrum.02800-23)

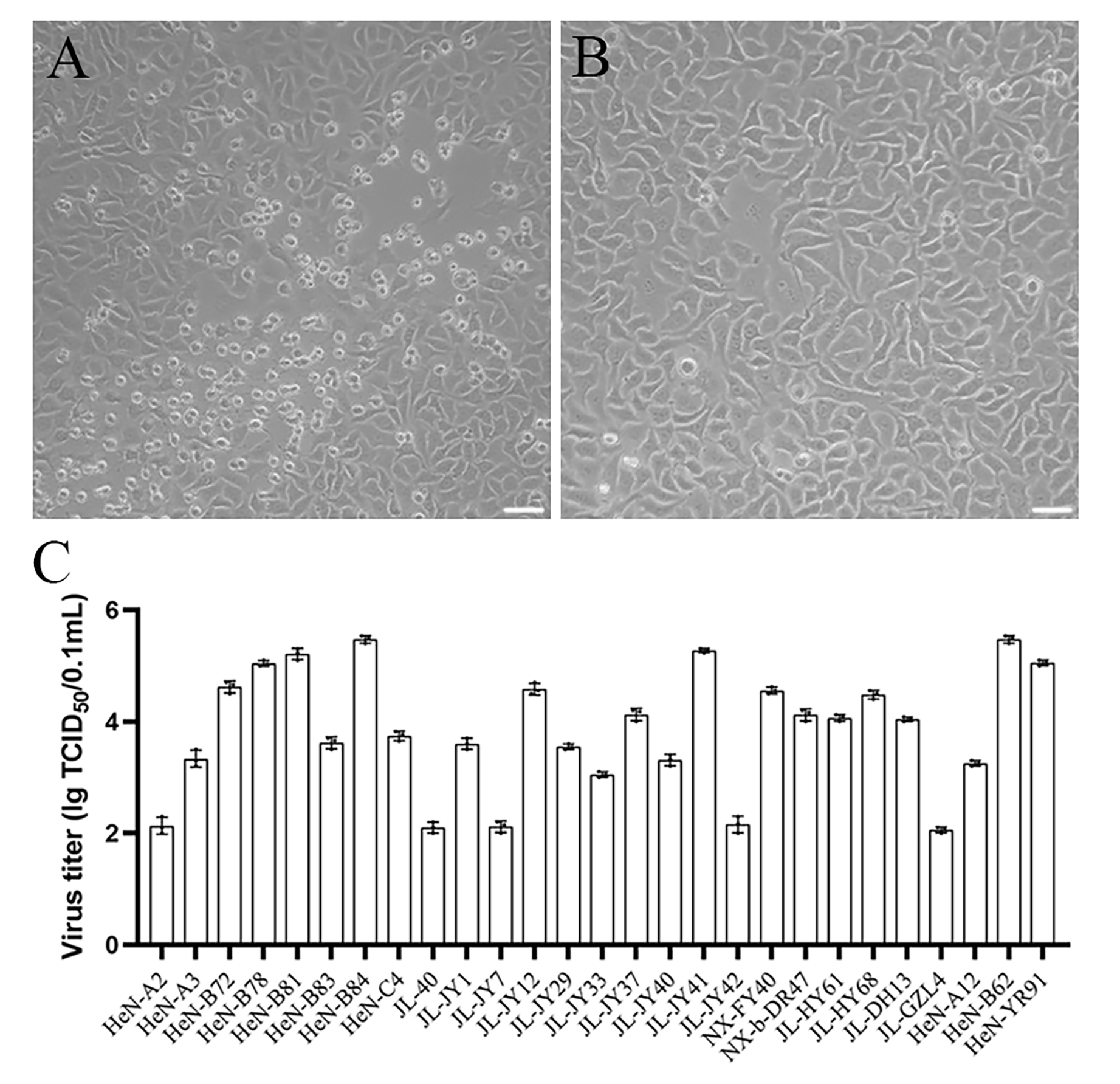

Supplement: Fig. S1 — Cytopathic effects (CPE) and growth characteristics analysis in Vero cells. [file spectrum.02800-23-s0001.tif]

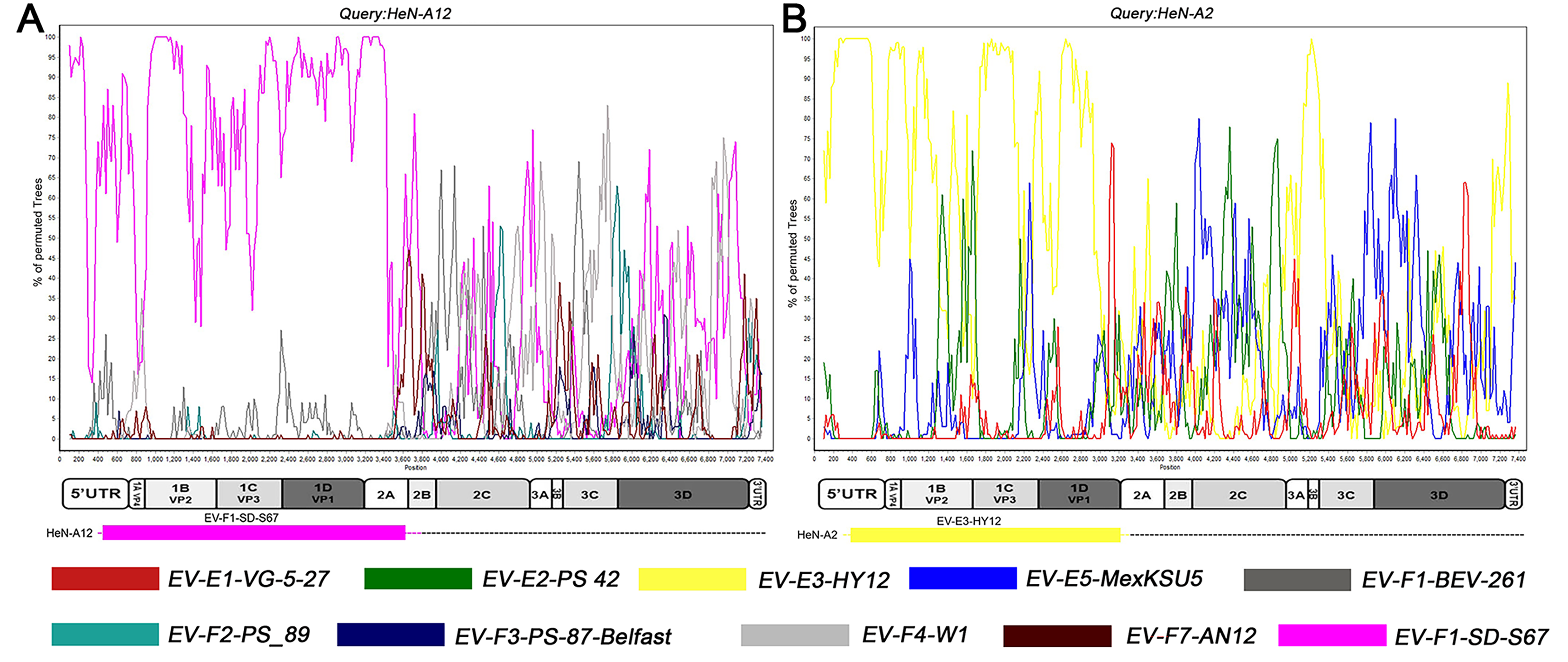

Supplement: Fig. S2 — Recombination analyses of HeN-A12 and HeN-A2 strains with EV-E and EV-F types. [file spectrum.02800-23-s0002.tif]

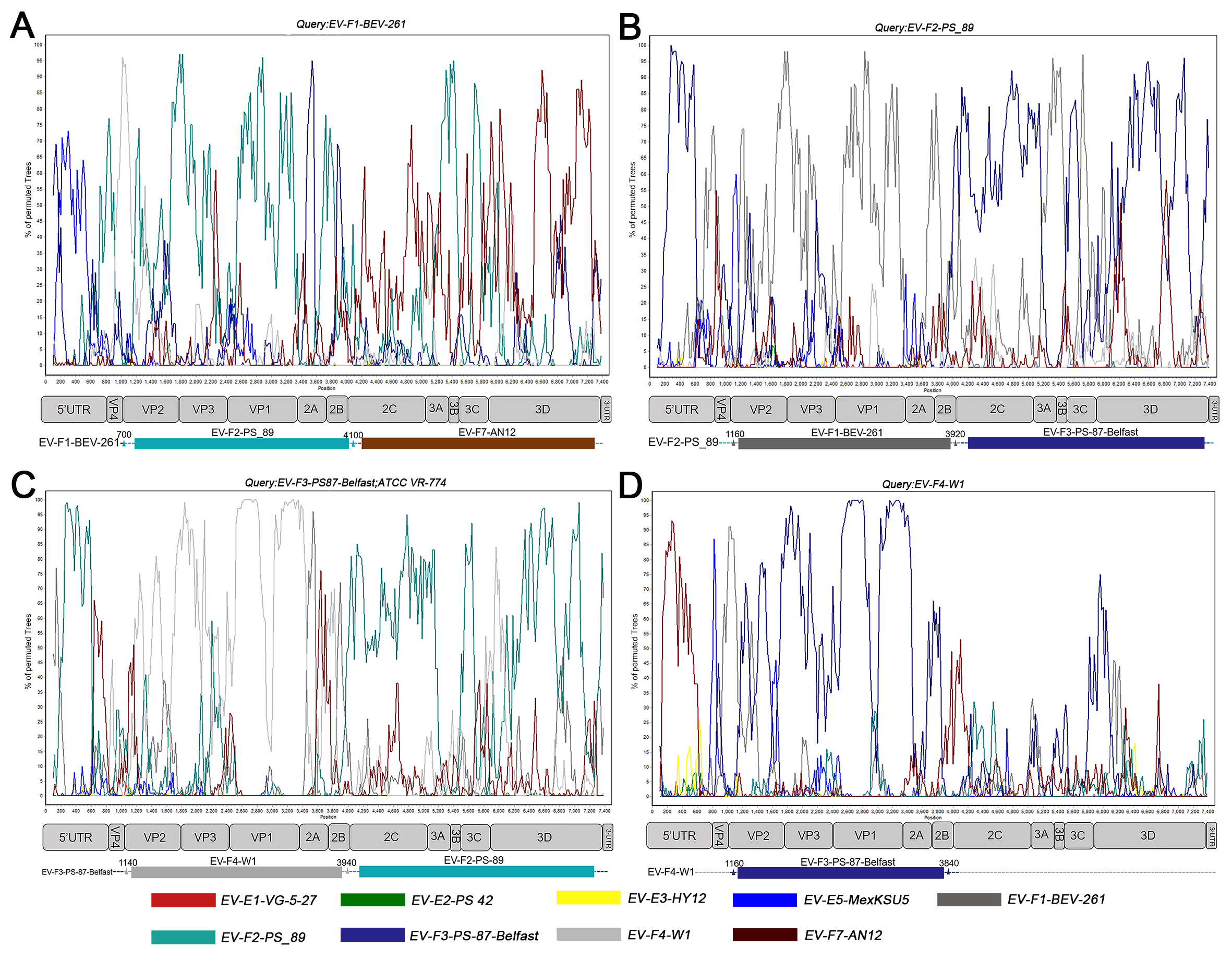

Supplement: Fig. S3 — Recombination analyses between EV-F subtypes. [file spectrum.02800-23-s0003.tif]
