# Supplementary material for: Genetic diversity and recombination of bovine enterovirus strains in China
Source: Microbiol Spectr. 2024 Feb 5;12(3):e02800-23. doi: 10.1128/spectrum.02800-23 (PMC10913430; doi:10.1128/spectrum.02800-23)
Supplement: Legends — Supplemental figure legends. [file spectrum.02800-23-s0004.docx]

**FIG S1** Cytopathic effects (CPE) and growth characteristics analysis in Vero cells. The CPE in Vero cells infected with representative novel bovine enterovirus strain JL-HY61 (A) and mock-infected Vero cells (B) were observed at 12 h postinfection (hpi). Compared to the normal cells, the cells infected by the isolated viruses showed a typical CPE including cell shrinkage, aggregation, roundness. The TCID_50_ of the novel bovine enterovirus strains were assayed and shown (C), which was calculated following a standard procedure based on the Reed-Muench method. Results are represented as mean ± SD of three independent experiments. Bar = 100 μm.

**FIG S2** Recombination analyses of HeN-A12 and HeN-A2 strains with EV-E and EV-F types. The genome sequences of HeN-A12 (A), and HeN-A2 (B) were used as query sequences in the bootscan analysis respectively. The structural map of bovine enterovirus genome was displayed below each panel. Different colored lines represent different reference strains. Each point plotted is the percentage identity within a sliding window 200 nt wide centered on the position plotted, with a step size of 20 nt between points.

**FIG S3** Recombination analyses between EV-F subtypes. The genome sequences of EV-F1-BEV-261 (A), EV-F2-PS_89 (B), EV-F3-PS-87-Belfast ATCC VR-774 (C), and EV-F4-W1 (D) were used as query sequences in the bootscan analysis. The structural map of bovine enterovirus genome was displayed below each panel. The possible crossover breakpoints were marked with rectangular box with the enterovirus subtypes and represented by corresponding colors. Each point plotted is the percentage identity within a sliding window 200 nt wide centered on the position plotted, with a step size of 20 nt between points.
