# Supplementary material for: Genetic diversity and recombination of bovine enterovirus strains in China
Source: Microbiol Spectr. 2024 Feb 5;12(3):e02800-23. doi: 10.1128/spectrum.02800-23 (PMC10913430; doi:10.1128/spectrum.02800-23)
Supplement: Table S1 — Primer sequences to amplify full-length sequences. [file spectrum.02800-23-s0005.docx]

| **TABLE S1** Primer sequences to amplify full-length sequences | | |
| --- | --- | --- |
| Name | Sequences (5'-3') | Position |
| EV-E-1-F | GCAGACCGGTTCGCTTAGGAC | 162~183 |
| EV-E-1-R | GTAGAACCGCTCGGCTGACACA | 1195~1216 |
| EV-E-2-F | CAACCGGCGGCTCAACCA | 873~890 |
| EV-E-2-R | ATCCGGCTCCAAAGGCATTACC | 3818~3839 |
| EV-E-3-F | TGGCGGTAATCTTGTTGGGTTCG | 3721~3743 |
| EV-E-3-R | GACGCGGCAGGACTATGATTGTGT | 5508~5531 |
| EV-E-4-F | TACGCCACGAAGAAGCCAGTTGTC | 5372~5395 |
| EV-E-4-R | GGATGTACCGGAGCAGCCAGATG | 6822~6844 |
| EV-E-3'-F | GGACGGTGAGCTTTTTGGTTTTGA | 6637~6660 |
| EV-E-6-F | TTTAAAACAGCCTGGGGGTTT | 1 ~ 22 |
| EV-E-6-R | ACGCGAGCAAGCCACCACATTA | 423~444 |
| EV-F-1-F | GGGCCGTGAATGCTGCTAATCC | 532~553 |
| EV-F-1-R | ACGCGTTGGCTGTGCTCATAAAAG | 3026~3049 |
| EV-F-2-F | AGACCGGGGCCACCTCAAATG | 2606~2626 |
| EV-F-2-R | TCGGCGACTGTAACTGGATGGATA | 4746~4769 |
| EV-F-3-F | GTCGCGTACACGGGGGTCTATGA | 3253~3276 |
| EV-F-3-R | AGCCGGTAAGTGTGCCTGGGTTTG | 6540~6563 |
| EV-F-4-F | CATTGAGGCGTCGAGTCTGAA | 6466~6486 |
| EV-F-4-R | CCATCCGGCGGGTGTATTG | 7375~7393 |
| EV-F-5-F | TTTTAAAACCAGCCTGGGGGTTG | 1~23 |
| EV-F-5-R | TTCCGCCTCCAACTTACG | 601~618 |
| EV-F-3'-F | ATTGCGTACGGTGATGATGTGAT | 6910~6932 |
