# Supplementary material for: Genetic diversity and recombination of bovine enterovirus strains in China
Source: Microbiol Spectr. 2024 Feb 5;12(3):e02800-23. doi: 10.1128/spectrum.02800-23 (PMC10913430; doi:10.1128/spectrum.02800-23)
Supplement: Table S2 — Reference strains for enterovirus. [file spectrum.02800-23-s0006.docx]

| **TABLE S2** Reference strains for enterovirus | | | | | | | | |  |
| --- | --- | --- | --- | --- | --- | --- | --- | --- | --- |
| Strains | Sequence | Accession No | Species | years | | country | | Host | |
| BEV IS1-Bos taurus-JPN-1990 | Complete | LC150009 | E | 1990 | Japan | | Bos taurus | |  |
| BEV10359 | Complete | MW959110 | E | 2021 | New Zealand | | bovine | |  |
| BEV-Egypt-2014 | Complete | KM667941 | E | 2014 | Egypt | | cattle | |  |
| BEV-GX1901 | Complete | MN607030 | E | 2019 | China | | cattle | |  |
| BEV-GX1902 | Complete | MN607031 | E | 2019 | China | | cattle | |  |
| BEV/JPN/TottoriU-31/2014 | Complete | LC081216 | E | 2014 | Japan | | Bos taurus | |  |
| BJ101 | Complete | MG650158 | E | 2015 | China | | bovine | |  |
| D 14-3-96 | Partial | DQ092786 | E | 1996 | Stendal | | Nasal swab of calf | |  |
| EV_NGR_2017 | Complete | MH719217 | E | 2017 | Nigeria | | bovine | |  |
| VG-5-27 | Complete | NC_001859 | E | 1957 | Northern Ireland | | calves | |  |
| PS 42 | Complete | DQ092792 | E | 1959 | Pennsylvania | | cow | |  |
| PAK-NIH-21E5 | Partial | JQ690741 | E | 2009 | Pakistan | | sewage samples | |  |
| MexKSU5 | Complete | KU172420 | E | 2015 | Mexico | | cattle | |  |
| LC-R4 | Complete | DQ092769 | E | 1957 | Michigan | | bovine | |  |
| NGR_2018 | Complete | MT542698 | E | 2018 | Nigeria | | – | |  |
| PA12-24791 | Complete | KC667561 | E | 2012 | USA | | bovine | |  |
| PD2017 | Complete | MG571548 | E | 2017 | Italy | | Bos taurus | |  |
| PS 83 | Complete | DQ092793 | E | 1958 | Pennsylvania | | cow | |  |
| SL305 | Complete | AF123433 | E | 1986 | Queensland | | – | |  |
| UFSM-SV89-91 | Complete | OL660538 | E | 1991 | Brazil | | cattle | |  |
| V251 | Complete | MT019688 | E | 2019 | Germany | | Bos taurus | |  |
| vir 404-03 | Complete | DQ092771 | E | 2003 | Arnsberg | | Intestine of calf | |  |
| W10_00309/293 | Complete | MW959111 | E | 2010 | New Zealand | | bovine | |  |
| W16_00544/CVGW76 | Complete | MW959106 | E | 2016 | New Zealand | | bovine | |  |
| 3A | Complete | AY508697 | F | 2003 | Maryland | | calves | |  |
| AN12 | Complete | NC_033695 | F | 2014 | Japan | | Bos taurus | |  |
| BEV Ho12-Bos taurus-JPN-2014 | Complete | LC150008 | F | 2014 | Japan | | Bos taurus | |  |
| BEV IS2-Bos taurus-JPN-1990 | Complete | LC150010 | F | 1990 | Japan | | Bos taurus | |  |
| BEV-261 | Complete | DQ092770 | F | 1958 | Washington | | bovine | |  |
| BHM26 | Complete | HQ917060 | F | 2008 | China | | bovine | |  |
| BJ001 | Complete | HQ663846 | F | 2009 | China | | bovine | |  |
| PS 89 | Complete | DQ092795 | F | 1957 | Pennsylvania | | cow | |  |
| PS87-Belfast;ATCC VR-774 | Complete | DQ092794 | F | 1962 | Pennsylvania | | calf | |  |
| W1 | Complete | AY462106 | F | 2002 | New Zealand | | Trichosurus vulpecula | |  |
| IL-alpaca | Complete | KC748420 | F | 2007 | USA | | Vicugna pacos (alpaca) | |  |
| NGR_2018-MN650196 | Complete | MN650196 | F | 2018 | Nigeria | | – | |  |
| PS87 | Complete | AY508696 | F | 2003 | United Kingdom | | bovine | |  |
| W6 | Complete | AY462107 | F | 2002 | New Zealand | | Trichosurus vulpecula | |  |
| W16_00059/89 | Complete | MW959104 | F | 2015 | New Zealand: Canterbury | | bovine | |  |
| W16_00059/139 | Complete | MW959105 | F | 2015 | New Zealand: Canterbury | | bovine | |  |
| W16_00544/CVGW336 | Complete | MW959107 | F | 2016 | New Zealand: King Country | | bovine | |  |
| W16_00544/Red6 | Complete | MW959108 | F | 2016 | New Zealand: King Country | | bovine | |  |
| W16_00634/15 | Complete | MW959109 | F | 2016 | New Zealand: King Country | | bovine | |  |
| W17_00094/318 | Complete | MW959112 | F | 2017 | New Zealand: King Country | | bovine | |  |
| W17_02174/23 | Complete | MW959113 | F | 2017 | New Zealand: Manawatu | | bovine | |  |
| S0098b/CA16/2013/CHN | Complete | KM402020.1 | A | 2013 | China | | Homo sapiens | |  |
| S0102b/EV71/2013/CHN | Complete | KM402021.1 | A | 2013 | China | | Homo sapiens | |  |
| Beijing-R0132 | Complete | KP240936.1 | D | 2014 | China | | Homo sapiens | |  |
| CEV-JL14 | Complete | NC_034267.1 | G | 2014 | China | | Capra hircus | |  |
| EV7-15936-01 | Complete | AY896765.1 | B | 2001 | Azerbaijan | | sewage | |  |
| EV30-8477-98 | Complete | AY896767.1 | B | 1998 | Russia | | Human with meningitis | |  |
| HY12 | Complete | KF748290.1 | E | 2012 | China | | Bovine | |  |
| K2577 | Complete | AF123432.1 | E | 1986 | Australian | | – | |  |
| N203 | Complete | AF414373.2 | J | 1972 | – | | simian | |  |
| US/KY/14-18953 | Complete | KM851231.1 | D | 2014 | USA | | Homo sapiens | |  |
| V2-Tol.1 | Complete | HQ738303.1 | C | 2005 | Madagascar | | Homo sapiens | |  |
| V3-Tul.7 | Complete | HQ738302.1 | C | 2005 | Madagascar | | Homo sapiens | |  |
| Sev-nj1 | Complete | KT581587.1 | J | 2013 | China | | primate | |  |
| 1715 UWB | Complete | NC_038309.1 | H | – | – | | Simian | |  |
| 19CC | Complete | NC_038310.1 | I | 2013 | United Arab Emirates: Dubai | | Dromedary | |  |
| rodent/Mc/PicoV/Tibet2015 | Complete | KX156159.1 | K | 2015 | China | | Rodent | |  |
| SEV-gx | Complete | NC_029905.1 | L | 2014 | China | | Macaca mulatta | |  |
| ATCC VR-1559 | Complete | NC_038311.1 | Rhinovirus A | – | ATCC | | Human | |  |
| ATCC VR-485 | Complete | FJ445112.1 | Rhinovirus B | – | ATCC | | Human | |  |
| HRV-QPM | Complete | EF186077.2 | Rhinovirus C | – | Australia | | Human | |  |
| rodent/Ee/PicoV/NX2015 | Complete | NC_038989.1 | K | 2015 | China | | rodent | |  |
| K2-TR2016 | Partial | MG702512.1 | E | 2016 | Turkey | | cattle | |  |
| EV-E/THAI/B1079 | Partial | MH894632.1 | E | 2016 | Thailand | | cattle | |  |
| B1-TR2016 | Partial | MG702506.1 | E | 2016 | Turkey | | cattle | |  |
| 114-TR2016 | Partial | MG702507.1 | E | 2016 | Turkey | | cattle | |  |
| BEV8/2021/CHN | Complete | ON932193.1 | E | 2021 | China | | cow | |  |
| GXYL2213 | Complete | ON584772.1 | E | 2022 | China | | cattle | |  |
| GXNN21X5 | Partial | OM654380.1 | E | 2021 | China | | Water buffalo | |  |
| Jena 38/02 | Partial | DQ092788.1 | E | / | Germany | | / | |  |
| SD 1182 II | Partial | DQ092784.1 | E | / | Germany | | / | |  |
| D 8/01 | Partial | DQ092782.1 | E | / | Germany | | / | |  |
| 56/59/1 | Partial | DQ092778.1 | E | / | Germany | | / | |  |
| E 6-82 | Partial | DQ092776.1 | E | / | Germany | | / | |  |
| VD 2860/1-99 | Partial | DQ092774.1 | E | / | Germany | | / | |  |
| D 58/96-V2130 | Partial | DQ092772.1 | E | / | Germany | | / | |  |
| EV-E/THAI/B1121 | Partial | MH894633.1 | E | 2016 | Thailand | | cattle | |  |
| EV-E/THAI/B2008 | Partial | MH894631.1 | E | 2016 | Thailand | | cattle | |  |
| EV-E/THAI/B2005 | Partial | MH894630.1 | E | 2016 | Thailand | | cattle | |  |
| EV-E/THAI/B1139 | Partial | MH894629.1 | E | 2016 | Thailand | | cattle | |  |
| EV-E/THAI/B1029 | Partial | MH894628.1 | E | 2016 | Thailand | | cattle | |  |
| B6-TR2016 | Partial | MG702511.1 | E | 2016 | Turkey | | cattle | |  |
| M1-TR2016 | Partial | MG702510.1 | E | 2016 | Turkey | | cattle | |  |
| M2-TR2016 | Partial | MG702509.1 | E | 2016 | Turkey | | cattle | |  |
| BOZ-TR2016 | Partial | MG702508.1 | E | 2016 | Turkey | | cattle | |  |
| 3065-TR2016 | Partial | MG702505.1 | E | 2016 | Turkey | | cattle | |  |
| 5741-TR2016 | Partial | MG702504.1 | E | 2016 | Turkey | | cattle | |  |
| K38-TR2016 | Partial | MG702503.1 | E | 2016 | Turkey | | cattle | |  |
| HLJ-3531/2013 | Partial | KJ956699.1 | E | 2013 | China | | bovine | |  |
| PAK_NIH_123E1 | Partial | JQ690747.1 | E | 2010 | Pakistan | | sewage sample | |  |
| PAK_NIH_92E1 | Partial | JQ690746.1 | E | 2010 | Pakistan | | sewage sample | |  |
| PAK_NIH_68E2 | Partial | JQ690745 | E | 2010 | Pakistan | | sewage sample | |  |
| PAK_NIH_67E2 | Partial | JQ690744 | E | 2010 | Pakistan | | sewage sample | |  |
| PAK_NIH_59E1 | Partial | JQ690743 | E | 2010 | Pakistan | | sewage sample | |  |
| PAK_NIH_48E3 | Partial | JQ690742 | E | 2011 | Pakistan | | sewage sample | |  |
| PAK-NIH-21E5 | Partial | JQ690741 | E | 2009 | Pakistan | | sewage sample | |  |
| W17_02174/75 | Complete | MW959114 | F | 2017 | New Zealand: Manawatu | | bovine | |  |
