# Supplementary material for: Genetic diversity and recombination of bovine enterovirus strains in China
Source: Microbiol Spectr. 2024 Feb 5;12(3):e02800-23. doi: 10.1128/spectrum.02800-23 (PMC10913430; doi:10.1128/spectrum.02800-23)
Supplement: Table S4 — Algorithms of the RDP4 package used to predict the recombination event. [file spectrum.02800-23-s0008.docx]

| **TABLE S4** Algorithms of the RDP4 package used to predict the recombination event | | | | | | | | |  |
| --- | --- | --- | --- | --- | --- | --- | --- | --- | --- |
| Recombinant strain | Parent major/minor | Model (average p-value) | | | | | | |  |
|  |  | RDP | BootScan | MaxChi | GENECONV | 3Seq | Chimaera | SiScan |  |
| HeN-B62 | EV-F3-PS-87-Belfast/EV-F7-AN12 | 1.33E-15 | 4.40E-10 | 1.65E-13 | - | 2,16E-06 | 1.34E-12 | 9.70E-37 | |
| EV-F7-AN12 | EV-F1-BEV-261/  EV-E5-MexKSU5 | 1.12E-21 | 3.73E-22 | 5.48E-25 | 7.15E-11 | 3.96E-12 | 5.21E-19 | - | |
| EV-E5-MexKSU5 | EV-E1-VG-5-27/  EV-F7-AN12 | 3.92E-28 | 2.57E-34 | 2.35E-16 | 3.25E-14 | 2.66E-14 | 1.05E-09 | 6.43E-18 | |
| HeN-A2 | HY12/ EV-E2-PS 42 | 2.20E-21 | 1.45E-24 | 1.58E-07 | 1.04E-18 | - | 6.75E-05 | 3.61E-14 | |
| HeN-A12 | SD-S67/HeN-B62 | 1.57E-27 | 2.24E-25 | 1.78E-24 | 9.68E-10 | 3.77E-03 | 3.81E-11 | 1.39E-53 | |
| EV-F1-BEV-261 | EV-F2-PS_89/  EV-F7-AN12 | - | 4.03E-04 | 2.02E-06 | 9.93E-01 | 1.80E-04 | 3.26E-05 | - | |
| EV-F2-PS_89 | EV-F3-PS-87-Belfast/ EV-F1-BEV-261 | 1.11E-04 | - | 3.42E-08 | - | 7.01E-06 | 9.11E-07 | - | |
| EV-F3-PS-87-Belfast | EV-F2-PS_89/  EV-F4-W1 | 1.55E-21 | 6.87E-13 | 2.64E-23 | - | 2.22E-16 | 3.77E-22 | - | |
| EV-F4-W1 | EV-F3-PS-87-Belfast/ EV-F7-AN12 | 6.21E-12 | 4.04E-07 | 7.29E-04 | - | 7.86E-05 | 6.39E-03 | 5.39E-19 | |
